# Development of a EST dataset and characterization of EST-SSRs in a traditional Chinese medicinal plant, *Epimedium sagittatum *(Sieb. Et Zucc.) Maxim

**DOI:** 10.1186/1471-2164-11-94

**Published:** 2010-02-08

**Authors:** Shaohua Zeng, Gong Xiao, Juan Guo, Zhangjun Fei, Yanqin Xu, Bruce A Roe, Ying Wang

**Affiliations:** 1 Key Laboratory of Pant Germplasm Enhancement and Speciality Agriculture, Wuhan Botanical Garden, Chinese Academy of Sciences, Wuhan, Hubei 430074, China; 2 The Graduate School, Chinese Academy of Sciences, Beijing, 100039, China; 3 Boyce Thompson Institute for Plant Research, Cornell University, Ithaca, NY 14853, USA; 4 USDA Robert W. Holley Center for Agriculture and Health, Ithaca, NY 14853, USA; 5 Stephenson Research and Technology Center, University of Oklahoma, Norman, Oklahoma 73019, USA

## Abstract

**Background:**

*Epimedium sagittatum *(Sieb. Et Zucc.) Maxim, a traditional Chinese medicinal plant species, has been used extensively as genuine medicinal materials. Certain *Epimedium *species are endangered due to commercial overexploition, while sustainable application studies, conservation genetics, systematics, and marker-assisted selection (MAS) of *Epimedium *is less-studied due to the lack of molecular markers. Here, we report a set of expressed sequence tags (ESTs) and simple sequence repeats (SSRs) identified in these ESTs for *E. sagittatum*.

**Results:**

cDNAs of *E. sagittatum *are sequenced using 454 GS-FLX pyrosequencing technology. The raw reads are cleaned and assembled into a total of 76,459 consensus sequences comprising of 17,231 contigs and 59,228 singlets. About 38.5% (29,466) of the consensus sequences significantly match to the non-redundant protein database (E-value < 1e-10), 22,295 of which are further annotated using Gene Ontology (GO) terms. A total of 2,810 EST-SSRs is identified from the *Epimedium *EST dataset. Trinucleotide SSR is the dominant repeat type (55.2%) followed by dinucleotide (30.4%), tetranuleotide (7.3%), hexanucleotide (4.9%), and pentanucleotide (2.2%) SSR. The dominant repeat motif is AAG/CTT (23.6%) followed by AG/CT (19.3%), ACC/GGT (11.1%), AT/AT (7.5%), and AAC/GTT (5.9%). Thirty-two SSR-ESTs are randomly selected and primer pairs are synthesized for testing the transferability across 52 *Epimedium *species. Eighteen primer pairs (85.7%) could be successfully transferred to *Epimedium *species and sixteen of those show high genetic diversity with 0.35 of observed heterozygosity (*Ho*) and 0.65 of expected heterozygosity (*He*) and high number of alleles per locus (11.9).

**Conclusion:**

A large EST dataset with a total of 76,459 consensus sequences is generated, aiming to provide sequence information for deciphering secondary metabolism, especially for flavonoid pathway in *Epimedium*. A total of 2,810 EST-SSRs is identified from EST dataset and ~1580 EST-SSR markers are transferable. *E. sagittatum *EST-SSR transferability to the major *Epimedium *germplasm is up to 85.7%. Therefore, this EST dataset and EST-SSRs will be a powerful resource for further studies such as taxonomy, molecular breeding, genetics, genomics, and secondary metabolism in *Epimedium *species.

## Background

Herb epimedii, a traditional Chinese medicinal herb, is prepared from the aerial parts of *Epimedium *species of Berberidaceae, which is a basal eudicot family containing enormous medicinal plants. *E. sagittatum *(Sieb. Et Zucc.) Maxim, together with other four *Epimedium *species, *E. brevicornu *Maxim, *E. pubescens *Maxim, *E. wushanense *T. S. Ying, and *E. koreanum *Nakai, is listed in Chinese Pharmacopoeia [1]. Herb epimediicontains various bioactive components and has been utilized extensively as a tonic and antirheumatic medicinal herb for thousands of years in China. Currently, Herb epimedii is still widely used to treat many diseases such as impotence, frequency/urgency of urination, coronary heart disease, chronic bronchitis, and neurasthenia [2]. In addition, *Epimedium *species are also used as ground cover plants and ornamental plants because of the abundance of flower colours and flower patterns. Therefore, *Epimedium *species possess enormous potentials for commercial application. The molecular research of *Epimedium*, however, is lagged behind. Firstly, at present, many studies focus on extracting bioactive components from *Epimedium *[3-5], but metabolic pathways involved in producing these bioactive components are still largely unknown. Secondly, many studies have attempted to distinguish taxonomically *Epimedium *species and detect the genetic diversity of *Epimedium *[6-8]. But only a few of genomic markers have been exploited, so far, in our lab for studying genetic diversity of natural populations, including 14, 19, and 12 SSRs for *E. sagittatum *[9], *E. koreanum *[10], and *E. brevicornu *[11], respectively. The most important thing is that, according to our knowledge, most genomic SSRs exploited fail to transfer in other *Epimedium *species. Thus, more markers are needed for in-depth understanding of the natural diversity and for developing strategies for sustainable utilization of *Epimedium*. Thirdly, although many papers reported taxonomic results of *Epimedium *using different molecular markers such as RAPD [12], RFLP [12], AFLP [13], and rDNA [6,7,14], the taxonomical relationships among *Epimedium *species, especially closely related Chinese species, are still not clear because of the shortcoming of molecular markers with little polymorphic information or the limited number of species sampled in former studies. Moreover, the morphological characteristics of *E. sagittatum *vary greatly among different populations, and it is still not known whether it belongs to polytypic species or a complex of cryptic species. Finally, according to our knowledge, the novel gene discovery and molecular breeding of *Epimedium *new cultivars haven't been started yet due to the lack of genetic and genomic information.

Generation of a large-scale expressed sequence tag (EST) dataset is a useful approach to accelerate the researches of non-model species, especially for *Epimedium*. As a valuable resource for comparative genomics, functional genomics and biodiversity study, EST database has been established for model plant species and non-model species (citrus, grape, kiwifruit, etc.). So far, only three Ranunculales species (*Aquilegia formosa × Aquilegia pubescens, Eschscholzia californica*, and *Papaver somniferum*) have EST sequences available in the public databases and very limited number of EST sequences are available for these species. Although two cDNA libraries have been constructed using flower and leaf materials of *E. brevicornu *[15,16], no EST sequences have been uploaded to National Center for Biotechnology Information (NCBI, http://www.ncbi.nlm.nih.gov) database yet. As of June 2009, only 398 nucleotide sequences are available for *Epimedium *in NCBI.

Comparing with other types of molecular markers, SSR marker has many advantages including high abundance, random distribution in the entire genome, high information content, codominant inheritance, and reproducibility. According to the original sequences used for identification of simple repeats, SSRs are divided in two categories, genomic SSRs derived from random genomic sequences and EST-SSRs derived from expressed sequence tags. It is very time-consuming, cost-expensive and labor-intensive for developing genomic SSRs from the repeat-enriched genomic library when compared with the development of EST-SSR. Genomic SSRs have neither genic function nor close linkage to transcriptional regions, while EST-SSRs are potentially tightly linked with functional genes that perhaps control certain important agronomic characters [17]. Therefore, EST-SSRs are very useful for MAS in plant breeding. Besides, EST-SSR markers contain high transferability because EST-SSRs are derived from expressed sequences that are more conserved than the non-genic sequences and can be found in other relative species [18]. Due to the sharp increase of EST sequences deposited in NCBI, more and more EST-SSRs have been identified and used extensively for comparative mapping, DNA fingerprinting, biodiversity, and evolutionary studies in lots of species [19,20]. So far, surveying the polymorphism, diversity and transferability of EST-SSRs have been performed in *Vitis *[18], *Citrus *[20], *Coffea *[21,22], *Medicago *[23], *Glycine *[24], *Phaseolus *[25], *Hordeum *[26], *Festuca *[27], and *Triticum *[28].

Here, we report the generation of a large-scale EST dataset from young leaves of *E. sagittatum *using the cost-effective 454 GS-FLX pyrosequencing technology, the development and characterization of a set of EST-SSRs, and the transferability of EST-SSRs across genus *Epimedium*. Therefore, the EST dataset can lay a foundation and facilitate MAS breeding and taxonomy in genus *Epimedium*, and decode secondary metabolism in *Epimedium *species.

## Results

### Contig assembly of 454 reads

A total of 228,768 sequences (51.7 Mb) are generated from leaf cDNAsof *E. sagittatum*. After removing SMART primers, polyA tail, and low quality sequences, a total of 217,380 high quality sequences is retained with a total length of 50.9 Mb and an average length of 224.9 bp ranging from 50 bp to 556 bp (Additional file 1). After clustering and assembly, 76,459 consensus sequences with a total length of 18.8 Mb (6.5 Mb of 17,231 contigs and 12.3 Mb of 59,228 singlets) are obtained (Additional file 1). The length of contigs ranges from 51 bp to 1,911 bp with an average of 375.9 bp, and that of singlets ranges from 50 bp to 556 bp with an average of 207.8 bp (Additional file 1).

The size distribution of *Epimedium *consensuses is shown in table 1. The length for the majority of ESTs is 201-300 bp, which is consistent with the 454 GS-FLX sequencing capacity. After the assembly, although the size distribution pattern of singlets is similar to that of whole EST dataset, there are 5,497 contigs with sequences longer than 400 bp (Table 1). In addition, about 7.2% of contigs are still less than 200 bp after assembly. This might be due to the short length of the individual read and/or the low coverage of the transcriptome represented in this dataset. Most of the consensuses are derived from few reads. For example, 7,509 (43.6% out of contigs) and 3,074 (17.8% out of contigs) consensuses are derived from 2 and 3 reads, respectively (Table 2).

**Table 1:** Size distribution of *E. sagittatum *ESTs before and after assembly

Items	1-100 bp	101-200 bp	201-300 bp	301-400 bp	>400 bp
Cleaned EST sequence	17,801	50,516	147,708	1,334	21
Singlet	6,720	14,171	37,444	875	18
Contig	163	1,084	5,461	5,026	5,497
Consensus	6,883	15,255	42,905	5,901	5,515

**Table 2:** Number of ESTs in the assembled consensus sequences

Number of reads per consensus	Number of consensuses
2	7,509(43.6%)
3	3,074(17.8)
4	1,600(9.3%)
5	1,029(6.0%)
6	667(3.9%)
7	499(2.9%)
8	385(2.2%)
9	267(1.5%)
10	192(1.1%)
11-15	688(4.0%)
16-20	352(2.0%)
21-25	204(1.1%)
26-30	138(0.8%)
31-35	94(0.5%)
36-40	81(0.5%)
41-45	60(0.3%)
46-50	43(0.2%)
>51	349(2.0%)

Note: the numbers in parentheses indicate the percentages of certain consensuses in all contigs.

### Structural and functional annotation of ESTs

**Figure 1:**
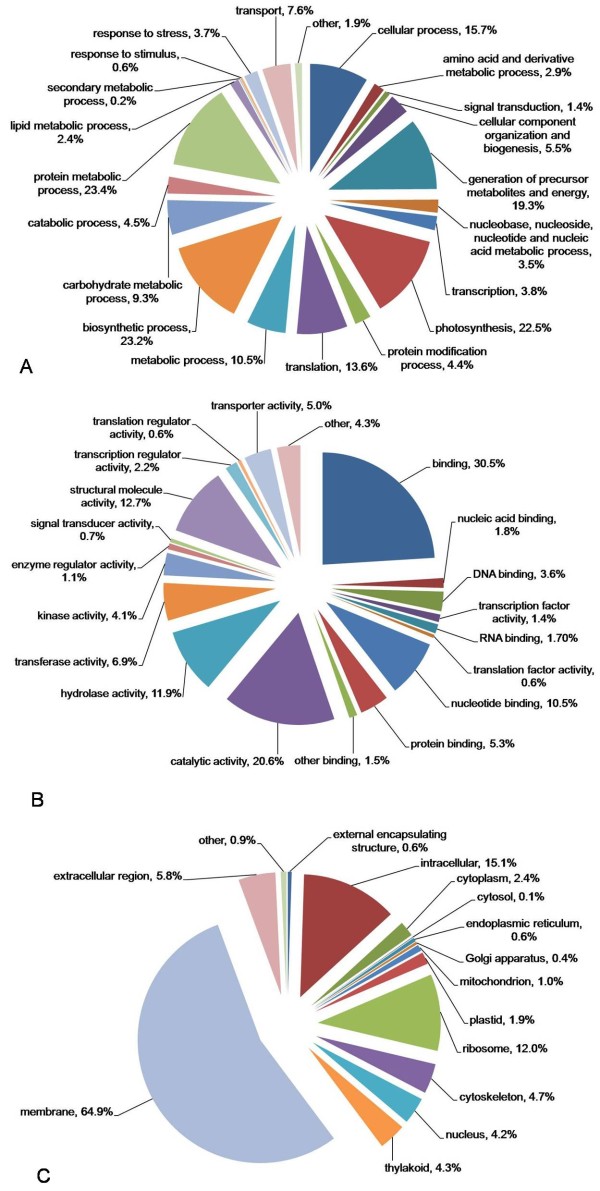
**Pie chart representations of GO-annotation results of 22,295 *E. sagittatum *consensus sequences**. The total numbers of *Epimedium *consensus sequences annotated for each category are 12,242 for Biological Process (A), 13,807 for Molecular Function (B), and 12,308 for Cellular Component (C). Since one gene product can be assigned to more than one GO terms, the total percentage in each category could excess 100%.

The consensus sequences are blasted against non-redundant protein database (NR database) and 29,466 consensuses comprising of 10,245 contigs and 19,221 singlets have significant hits to NR database (Additional file 1). All the consensus sequences are also compared against Pfam domain database and about 50% (38,220) of them are successfully annotated. Gene Ontology (GO) terms are further assigned to 38,220 *Epimedium *consensus sequences that are successfully annotated based on Pfam matches. Only 22,295 *Epimedium *consensus sequences are able to be assigned GO numbers. Figure 1 shows the percentage distributions of gene ontology terms according to the GO consortium. Protein metabolic process (23.4%) is the most dominant group out of 12,242 consensus sequences that are annotated to the biological process category. It is followed by the biosynthetic process at 23.2%, photosynthesis at 22.5%, generation of precursor metabolites and energy at 19.3%, cellular process at 15.7%, translation at 13.6%, metabolic process at 10.5%, and carbohydrate metabolic process at 9.3% (Figure 1A). A total of 13,807 sequences could be assigned to the molecular function category. Binding (30.5%) is the most dominant group followed by catalytic activity (20.6%), structural molecular activity (12.7%), hydrolase activity (11.9%), nucleotide binding (10.5%), transferase activity (6.9%), and transcription regulator activity (2.2%) (Figure 1B). With regard to the cellular component, 64.9% of 12,308 consensus sequences are assigned to membrane followed by intracellular (15.1%), ribosome (12.0%) and extracellular region (5.8%) (Figure 1C).

Being famous medicinal plants used in China, *Epimedium *species have flavonoids as the major phytochemical components. There are also many other unidentified compounds in *Epimedium *species [29]. Thus, genes related to secondary metabolism, especially for the flavonoid biosynthesis, are under further studies in order to decipher the molecular mechanism of natural variation of phytochemical components and facilitate the molecular breeding of medicinal cultivars. In our EST collection, 29 EST consensus sequences are annotated relating to secondary metabolic process (Figure 1A), including genes encoding key enzymes in the flavonoid biosynthetic pathway such as phenylalanine ammonia-lyase, cinnamate-4-hydroxylase, 4-counaroyl:ligase CoA, chalcone synthase, chalcone isomerase, flavanone 3 hydroxylase, flavanone 3' hydroxylase, flavanone 3'5' hydroxylase, flavonol synthase, flavone synthase, dihydroflavonol 4-reductase, anthocyanidin synthase, rhamnosyl transferase, and UDPG-flavonoid glucosyl transferase. In addition, more than 300 EST consensus sequences were annotated relating to transcription regulator activity (Figure 1B), part of which might play roles in regulating flavonoid biosynthesis and metabolism. For example, regulatory genes are annotated as members of WD40 family, MYB family, and bHLH family that can form a triplex compound to regulate the specific expression patterns of flavonoid structural genes. All these structural and regulatory genes are being cloned and functional studies are carrying out in our lab. Further studies such as molecular modification (or gene transformation) and metabolic engineering of enzymes are also in progress.

### Frequency and distribution of EST-SSRs in the *Epimedium *transcriptome

A total of 2,629 sequences containing 2,810 SSRs have been identified from 76,459 consensus sequences, with 167 EST sequences containing at less two SSRs. The frequency of EST-SSRs observed in *Epimedium *transcriptome is 3.67%, and the distribution density is 149.5 per Mb. The occurrence of different repeat type is shown in Table 3. The most abundant repeat type is trinucleotide (55.2%, 1,552) followed by dinucleotide (30.4%, 855), tetranucleotide (7.3%, 204), hexanucleotide (4.9%, 137), and pentanucleotide repeat unit (2.2%, 62). The frequencies of EST-SSRs distributed in different repeat numbers are also shown in Table 3. There are 1,063 SSRs with 5 tandem repeats, which is the most common repeat number (37.8%) followed by 6 tandem repeats (21.2%, 595), 4 tandem repeats (11.9%, 333), 7 tandem repeats (9.8%, 274) and 8 tandem repeats (7.5%, 210). The dominant repeat motif is AAG/CTT with a frequency of 23.6% (663), followed by AG/CT (19.3%, 543), ACC/GGT (11.1%, 312), AT/AT (7.5%, 211), and AAC/GTT (5.9%, 165) (Table 4). More details about different repeat motif of di- and trinucleotide repeats in EST-SSRs are listed in table 4. It is interesting that there is no CG/GC repeat motif and very few CCG/CGG repeats in our results.

**Table 3:** Frequencies of repeat type with repeat numbers in EST-SSRs from *E. sagittatum*

Motif length	Repeat number								total	%
			
	4	5	6	7	8	9	10	>10		
Di	-	-	234	171	147	78	58	167	855	30.4
Tri	-	1,010	353	99	58	11	12	9	1,552	55.2
Tetra	156	41	4	1	2	0	0	0	204	7.3
Penta	53	5	1	1	2	0	0	0	62	2.2
Hexa	124	7	3	2	1	0	0	0	137	4.9
total	333	1,063	595	274	210	89	70	176	2,810	
%	11.9	37.8	21.2	9.8	7.5	3.2	2.5	6.3		

**Table 4:** Frequencies of different repeat motifs of di- and trinucleotide repeats in EST-SSRs from *E. sagittatum*

Repeat motif	Repeat number							total	%
			
	5	6	7	8	9	10	>10		
AG/CT	-	128	103	103	56	43	110	543	19.3
AT/AT	-	47	52	33	15	11	53	211	7.5
AC/GT	-	59	16	11	7	4	4	101	3.6
AAG/CTT	384	171	47	39	7	9	6	663	23.6
ACC/GGT	234	55	11	10	2		0	312	11.1
AAC/GTT	116	23	18	5	1	1	1	165	5.9
AGC/CGT	58	25	9	1			0	93	3.3
ACG/CTG	51	16	3	1			0	71	2.5
AGG/CCT	37	26	4				0	67	2.4
AAT/ATT	47	14	3	1			0	65	2.3
ACT/ATG	45	7	2	1		1	1	57	2
AGT/ATC	35	16	2		1	1	1	56	2
CCG/CGG	3							3	0.1

### The *Epimedium *EST-SSR Transferability

**Figure 2:**
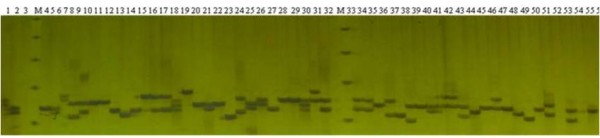
**Polyacrylamide gel electrophoresis of PCR fragments amplified by EsESP30 primer pairs**. Lane 1- 55 corresponded to the species listed in additional file 3. M indicated 25 bp ladder marker.

A total of 32 primer pairs (Additional file 2), referred to EsESP01 to EsESP32, have been stochastically selected among the 2,810 microsatellites to amplify 10 dinucleotide SSRs and 22 trinucleotide SSRs for testing the transferability in other *Epimedium *species (Additional file 3). Of these primer pairs, seven primer pairs (EsESP1, EsESP3, EsESP4, EsESP20, EsESP24, EsESP31, and EsESP32) cannot amplify any fragment in *E. sagittatum*, suggesting that these primers are not well designed. Four primers, EsESP14, EsESP19, EsESP26, and EsESP27, amplify numerous non-target bands, suggesting these primers are also problematic. Three primer pairs, EsESP05, EsESP25, and EsESP29, succeed in transferring to other *Epimedium *species, but amplify PCR products larger than the expected sizes. The rest of primer pairs produces polymorphic bands in most *Epimedium *species as shown in figure 2. Therefore, 85.7% (18 out of 21) EST-SSR primers can be transferred successfully to other *Epimedium *species. In theoretically, there are 56.2% (18/32) of 2810, namely ~1580, *E. sagittatum *EST-SSRs markers suitable for genetic studies in genus *Epimedium*.

Species from other genera, including *Vancouveria hexandra*, *Nandina domestica *Thunb., *Berberis julianae *Schneid., *Berberis sargentiana *Schneid., *Dysosma versipellis *(Hance) M. Cheng ex Ying, *Mahonia bealei *(Fort.) Carr., and *Mahonia fortunei *(Lindl.) Fedde., have been used to test the transferability of *Epimedium *EST-SSRs across different genus of Berberidaceae. A total of 8 primers, EsESP6, EsESP7, EsESP9, EsESP10, EsESP11, EsESP13, EsESP23, and EsESP28, can produce PCR fragments in *V. hexandra *(the most closely related genus reported by Wang et al [30]) with similar sizes and same SSR motifs as those in *Epimedium *(sequence data not shown). Only four primers, EsESP8, EsESP13, EsESP18, and EsESP21, can amplify PCR fragments in other Berberidaceae species mentioned above. However, sequences of the PCR fragments from genera *Berberis*, *Nandina*, *Dysosma*, and *Mahonia*, do not contain the SSR motifs found in *Epimedium *species (data not shown). Therefore, *Epimedium *EST-SSRs can successfully transfer to *Vancouveria*, but not other Berberidaceae genera tested.

### The genetic diversity and dendrogram in genus *Epimedium*

A total of 55 individuals representing 52 species (Additional file 3) have been used to evaluate the genetic diversity in genus *Epimedium*. Two primer pairs, EsESP07 and EsESP08, detect a single band in some species while null in other species. Thus, they are excluded for further study and the remaining 16 EST-SSRs are used to estimate the genetic diversity among 52 species. A high level of genetic diversity in genus *Epimedium *is discovered (Additional file 2). The number of alleles per locus ranges from 3 to 27 with an average of 11.9 alleles. *Ho *ranges from 0.04 to 0.6 with an average of 0.35. *He *ranges from 0.17 to 0.94 with an average of 0.65. Polymorphism information content (PIC) is from 0.17 for EsESP22 locus to 0.93 for EsESP21 locus. The observed length of PCR products vary greatly in a large spectrum compared with the fragment size in *E. sagittatum *(Additional file 2). The size variation of amplicons reflects the length of repetitive sequences in each SSR locus, suggesting that the polymorphism might be related to the differences of tandem repeat numbers (Figure 2).

In order to study the evolutionary relationship among *Epimedium *species, those 16 EST-SSR primers successfully transferring in almost all *Epimedium *species are used for reconstructing *Epimedium *phylogeny. *V. hexandra *is used as an outgroup species, because it is more closely related to *Epimedium *genus than *Jeffersonia dubia *(Maxim.) Benth. et Hook. f. and *Podophyllum peltatum *[30]. Furthermore, sequences of some loci in *V. hexandra *amplified by *Epimedium *EST-SSR primers confirm that similar single nucleotide repeats exist in *V. hexandra *as mentioned above. As shown in additional file 4, the phylogenetic relationship among all 52 species are supported by low bootstrap values, especially for Chinese species, partially due to the small number of EST-SSRs and the nature of EST-SSR with low PIC used in this study. Previous studies also found it is hard to differentiate the closely related Chinese species using different types of molecular markers including RAPD [12], RFLP [12], AFLP [13], and rDNA [6,7,14]. Therefore, more EST-SSR markers or combining results of different types of markers would provide more opportunities for better resolving the phylogeny among *Epimedium *species, which is an on-going project in our lab.

## Discussion

### *E. sagittatum* ESTs annotation

Currently, numerous studies are focusing on isolating the *Epimedium *bioactive components [3-5], but the potential molecular mechanism producing bioactive components is still unclear. Furthermore, little or no information regarding to its biological conservation, population genetics, molecular breeding, and molecular biology is reported for *Epimedium *species due to the lack of background information. The 454 GS-FLX pyrosequencing technology is a very cost-effective method to obtain large-scale EST sequences and will speed up the researches of less-studied *Epimedium *species. The technology has been broadly used for bacteria *de novo *sequencing, cDNA sequencing, small RNA sequencing, metagenomic sequencing and whole genome shotgun survey http://www.454.com/news-events/publications.asp. Margulies *et al*. [31] demonstrated that the technology had 100 fold sequencing capability at the cost of relative low accuracy than Sanger-based capillary electrophoresis sequencing systems when the GS20 sequencing machine was used. Recently, the new generation machine, GS-FLX, possesses more powerful sequencing capability of up to 200-300 bp per read and higher sequencing accuracy than that of GS20 http://www.454.com. In this study, an *Epimedium *transcriptome population is sequenced by 454 GS-FLX system, and a total of 226,544 sequence reads are generated. After assembly, 76,459 consensus sequences are obtained.

It is noticeable that only 29,466 *Epimedium *consensuses have significant hits in the NR protein database (Additional file 1). Thus, there are a large number of sequences that don't have any significant match in other species (46,993). This might be due to the following reasons: (1) the shortness of sequence reads leading to assemble difficultly, (2) inefficiency of blast using short sequences and the incompleteness of the known database, (3) the advantages of deep sequencing, which can discover novel genes with low expression levels, (4) the shortness of sequence reads resulting in low efficiency of annotation, which was confirmed by correlation test between sequence length and percentage of sequences successfully annotated (Additional file 5), and (5) part of consensuses representing UTRs. With regard to the first point, the size of raw reads ranged from 50 to 556 bp with an average length of 224.9 bp. After assembly, most contigs still range from 201 to 300 bp, and the average length only increased up to 245.7 bp. It is also supported by the fact that most contigs were assembled with 2-5 reads (Table 2). Thus, the shortness of sequences is the main disadvantage in this scenario when using 454 pyrosequencing technologies. Concerning on the second point, short query sequences might not be able to have sequence matches with a significant low E-value and high score, thus resulting in a high level of false negative results. In addition, Ranunculales is much less studied when compared with core eudicots and monocots, suggesting that a lot of Ranunculales lineage-specific genes might not be included in the database. As to the third point, EST fragments are directly sequenced instead of being cloned and sequenced, which leads to the truly random sequencing of all the expressed genes and might facilitate the discovery of rare genes. Emrich *et al *[32] found that coupling of LCM (Laser Capture Microdissection) and 454 sequencing technologies could facilitate the discovery of rare, possibly cell-type-specific transcripts. In summary, the nature of shortness in reads generated by 454 GS-FLX sequencing technology is the origin of low efficiency of annotation. However, it is still possible that new genes and/or Ranunculales- or even *Epimedium*- specific novel sequences could be discovered due to large-scale sequencing.

### Potential applications of *Epimedium *EST dataset

Combining all the available information, we have a set of 22,295 GO-annotated *Epimedium *consensus sequences, which is very valuable and cost-effective for a non-model organism by sequencing one tissue at certain developmental stage. Additionally, 10.5%, 9.3%, 3.0%, and 0.2% of 12,242 *Epimedium *GO-annotated consensuses for biological processes are related to metabolic process, carbohydrate metabolic process, amino acid and derivative metabolic process, and secondary metabolic process, respectively (Figure 1A). Many bioactive components of *Epimedium *might derive from these biological processes (Figure 1A). A total of 13,807 *Epimedium *consensuses is annotated for molecular function, 2.2% and 20.6% of which have transcription regulator activity and catalytic activity, respectively, suggesting that these genes might take part in regulating and catalyzing the production of bioactive components (Figure 1B). All of these sequences can provide fundamental information for the metabolic-engineering and molecular breeding of *Epimedium*.

In details, this large EST dataset of *Epimedium *is a powerful resource for further studies. Firstly, it can facilitate cloning genes of interest, especially for genes involved in controlling metabolic pathways such as the flavonoid pathway. The *Epimedium *EST dataset generated in our study will provide enough sequence information for designing primers to obtain full-length genes. Therefore, these sequences will promote the molecular study of secondary metabolism in medicinal *Epimedium *plants. Secondly, molecular markers can be exploited based on EST sequences for researches in the fields of biodiversity, taxonomy, and population genetics. Although genomic SSRs have been developed from *Epimedium*, they have much lower transferability among different *Epimedium *species than EST-SSRs [9-11]. Therefore, EST-SSRs can further promote the taxonomic study of *Epimedium *species, especially those closely related Chinese species. Thirdly, it is noticeable that some EST-based molecular markers might be linked to genes involved in controlling traits that are important for market value [17,33]. Thus, EST-SSRs can provide a powerful tool for genetic mapping and MAS. In summary, the EST dataset reported here can be used for decoding the molecular mechanism of the flavonoid pathway, as well as lays a foundation for biodiversity, population genetics, genomics, and functional genomics of *Epimedium *species.

### EST-SSR frequency and distribution in *Epimedium *transcriptome

A total of 2,810 perfect microsatellites over 12 bp have been identified from *Epimedium *EST dataset, i.e. 3.67% EST sequences possess single sequence repeats. Obviously, the frequency of SSRs detected in *Epimedium *is similar to that of other dicotyledonous species ranging from 2.65% to 16.82% [34]. The EST-SSR frequency is dependent on the following factors. Firstly, parameters used in exploring microsatellites affect the results dramatically, such as the repeat length threshold and the number of repeat unit. In the present study, mononucleotide repeat motifs are excluded in our strategy for identifying microsatellites. Secondly, the genome structure or composition may also account partially for the EST-SSR frequency, because the genetic-biochemical background of the cells plays important roles in fixation of *de novo*-generated SSRs [35]. Thirdly, different softwares used in detecting SSRs might also affect the frequency. Some SSR identifying tools can find out imperfect SSR, for example, Sputnik [36], while others can only identify perfect SSRs such as SSRIT [37] and MISA [20,21,26].

Theoretically, the frequency of di-, tri-, tetra-, penta-, and hexanucleotide repeats should be in turn decreased. However, trinucleotide repeat unit is the most dominant SSRs, followed by di-, tetra-, hexa-, and pentanucleotide repeat units (Table 3). It is consistent with the results reported for other plant species [20,23,38-40]. As shown in table 4, the most dominant di- and trinucleotide repeat motifs are AG/CT (19.3%) and AAG/CTT (23.6%), respectively. These results are in agreement with that of Morgante *et al *[36], Kumpatla *et al *[34], and Toth *et al *[35]. Interestingly, there are only three CCG/CGG trinucleotide repeats in *Epimedium*, which is the most predominant in monocots [36,38]. Our results strongly support and extend the notion of rarity of the CCG/CGG repeat units in a large number of dicotyledonous plants (*Citrus*, *Coffea*, *Medicag*, *Glycine*, etc.) [34]. These might be resulted from the high GC content and consequent codon usage bias in monocots [36,38]. Selection might also be against the CCG/CGG repeat unit because of the requirements of the splicing machinery [41]. Long CCG/CGG sequences could compete splicing machinery components and give rise to inadequate splicing. Moreover, CCG/CGG repeats, might form potential higher structures such as hairpin and quadruplex, and thus affect the efficiency and accuracy of splicing and influence the formation of mature mRNA [35,42].

### Primer designing for EST-SSRs

In this study, attempt of diagnosing transferability have been performed by 32 EST-SSR primer pairs designed according to *E. sagittatum *ESTs. Unfortunately, seven and four primer pairs fail to transfer in *Epimedium *and basal eudicot, respectively, which might be due to primer(s) across splice sites, the presence of large introns in genomic sequence, or primer(s) derived from a chimeric or questionable consensuses sequence [39]. Some primer pairs succeed in amplifying in some species while fail in other species, suggesting that null alleles exist. Null alleles might be resulted from some mutations, including the deletion of microsatellites, and indels or substitution in primer binding sites [39]. In addition, the observed sizes of PCR products in *Epimedium *species deviate frequently from the expected product sizes according to the EST sequences. For instance, polymorphic bands amplified by EsESP05, EsESP25, and EsESP29 are larger than the expected sizes. It might be due to the presence of introns in the corresponding genomic regions [27,39] and/or variation in the repeat numbers.

### The EST-SSR transferability in genus *Epimedium*

In the present study, transferability of EST-SSRs derived from *E. sagittatum *is high (85.7%) across 52 species in genus *Epimedium*. The high transferability of EST-SSRs is also reported in other plants. For instance, 90% EST-SSRs in *Citrus clementia *can be transferred successfully to ten *Citrus *species and three related genera [20], and 89% EST-SSRs in *Medicago truncatula *succeed in transferring to eight genotypes [23]. About 92% *Festuca arundinacea *EST-SSR primer pairs can produce characteristic SSR bands in at least one of the six related species [27]. Numerous studies have also demonstrated that EST-SSRs possess higher transferability due to the conservation characteristics of EST-SSRs when compared with SSRs derived from genomic libraries [43,44]. In genus *Epimedium*, the transferability of genomic SSRs derived from *E. sagittatum *[9] and *E. koreanum *[10] ranges from 21.4% to 42.9% and from 42.1% to 47.4%, respectively. Genomic SSRs isolated from *E. brevicornu *also possess low transferability ranging from 33.3% to 66.7% across other four medicinal *Epimedium *species [11]. Comparing with the high transferability of EST-SSRs (85.7%), the transferability of *Epimedium *genomic SSRs is remarkably lower.

## Conclusions

A large-scale EST dataset with 76,459 consensuses derived from *E. sagittatum *is reported in this study. A total of 22,295 sequences has been successfully annotated with GO terms based on the known sequences, and part of unique sequences are involved in the flavonoid metabolic pathway, which would facilitate deciphering the molecular mechanism of secondary metabolism in *Epimedium*. Based on this EST dataset, 2,810 are exploited by MISA software and 32 primer pairs are randomly selected for detecting transferability. The transferability of *E. sagittatum *EST-SSRs is up to 85.7% and there are ~1580 EST-SSR markers transferable in genus *Epimedium *for further genetic studies. *Ho *and *He *range from 0.04 to 0.6 and from 0.17 to 0.94 with an average of 0.35 and 0.65, respectively, and the number of alleles per locus ranges from 3 to 27 with an average of 11.9 alleles. These results suggest that the major germplasm of *Epimedium *show high genetic diversity. Due to the nature of EST-SSRs with high transferability and genomic SSRs with high polymorphism and both locating in different regions across whole genomes, combination of EST-SSRs derived from this study and genomic SSRs developed in our lab will be a powerful resource for molecular taxonomic study in genus *Epimedium *and constructing genetic maps. In addition, EST-SSRs tend to concentrate in the gene-rich regions, suggesting that part of EST-SSRs can be exploited for the use of MAS benefiting for *Epimedium *molecular breeding in the future. Therefore, the set of EST-SSRs developed in the present study is a promising resource for the related studies of *Epimedium*. In a word, *Epimedium *EST dataset is a valuable resource for studies in the fields of taxonomy, MAS, and secondary metabolism in genus *Epimedium*.

## Methods

### Plant materials

A total of 52 *Epimedium *species including 55 individuals (Additional file 3) were selected and investigated for the transferability of EST-SSRs derived from *E. sagittatum*. Leaf samples were collected from Wuhan Botanical Garden (Wuhan, Hubei Province, China) and Garden Vision Nursery (Boston, MS, USA). According to the taxa system reported by Stearn [45], samples cover two subgenera and three sections, and represent most of *Epimedium *germplasm.

### cDNA preparation

The red-magenta fully expanded leaves of *E. sagittatum *Maxim. grown in Wuhan Botanical Garden, Chinese Academy of Sciences, were harvested and prepared for RNA isolation. Total RNA was isolated according to the instruction of TRIzol kit (Invitrogen) and then purified to exclude the tRNA and rRNA and to enrich mRNA, according to the instruction of mRNA purification kit (Promega). mRNAs were reverse-transcribed by Powerscript™ II (Takara) with PCR primers SMART IV™ Oligonucleotide (5'-AAGCAGTGGTATCAACGCAGAGTGGCCATTACGGCCGGG-3') and CDS III/3' PCR Primer (5'-ATTCTAGAGGCCGAGGCGGCCGACATG-d(T)30_N-1_N-3'). Long Distance PCR for double strand cDNA amplification was performed with LA Taq enzyme (Takara) for 25 cycles (95°C for 30 s, 68°C for 8 min) according to the SMART™ cDNA Library Construction Kit User Manual. Finally, double strand cDNAs were purified using DNA purification kit (Qigen) to generate high quality cDNAs.

### EST processing, assembly, and annotation

Around 10 μg cDNAs were used for a half-plate sequencing run using 454 GS-FLX pyrosequencing technology. The sequencing was done at the Oklahoma University genome center. Considering the shortness of reads generated by 454 sequencing technology, full-length cDNA was sheared stochastically prior to sequence in order to randomly represent all transcripts. Sequence data for *Epimedium *Short Read Archive (SRA) described in this paper can be found at the public database [NCBI: SRA008151]. A total of 228,768 raw sequences was obtained. Lucy software [46] with default settings, except that minimum good sequence length cut-off was set to 50 bp, was used to remove low quality regions. SeqClean software http://compbio.dfci.harvard.edu/tgi/software/ was then used to eliminate poly A/T sequences, vector sequences, adaptor sequences and bacterial sequences. The resulted high quality sequences were assembled to consensus sequences using CAP3 software [47] with parameters as -p 95 -y 15 -s 251 -o 25. All consensus sequences were compared against Pfam database for protein domain analysis [48]. Based on the domain annotation, GO accessions of the consensus sequences were successfully assigned using Pfam2 go conversion file from the GO consortium http://www.geneontology.org/external2 go. GO annotations were formatted for input into the GOSlim program and the output was parsed to count the occurrence of each GO category.

### EST-SSR detection and primer designing

The EST-SSR detection was performed using Perl program MISA http://pgrc.ipk-gatersleben.de/misa. Since it was hard to distinguish real mononucleotide repeats and single nucleotide stretch error generated by 454 sequencing, mononucleotide repeats were excluded from this study. The parameters were designed for identifying perfect di-, tri-, tetra-, penta-, and hexanucleotide motifs with a minimum of 6, 5, 4, 4, and 4 repeats, respectively. Primer 3 software [49] was used to design primers. The major parameters for designing the primers were set as follows: primer length ranging from 18 bases to 28 bases with 20 as the optimum, PCR product size ranging from 100 to 300 bp, optimum annealing temperature 60°C, and GC content from 40% to 70% with 50% as optimum. According to these parameters, 32 primer pairs (Additional file 2) were synthesized and were used to detect the transferability in genus *Epimedium*.

### Survey of EST-SSR polymorphism

Total DNA was isolated from leaf samples according to the CTAB method [50]. PCR amplifications were carried out in a final volume of 10 μL containing 50 ng of genomic DNA, 1× PCR buffer [10 mM Tris-HCl (pH 8.4), 1.5 mM MgCl_2_], 0.2 mM dNTPs, 0.2 μM of each primer, and 0.5 U Taq polymerase (Biostar). The PCR reactions were performed under standard conditions for all primers in a thermocycler (Eppendorf). The annealing temperature was fixed for all primer pairs at 60°C. After 5 min at 95°C, 35 cycles were carried out with 30 s at 95°C, 30 s at 60°C, 40 s at 72°C for extension, and a final extension step of 10 min at 72°C. The separation of alleles was performed on a 6% polyacrylamide gel. PCR products were mixed with an equal volume of loading buffer. The mixture was denatured at 95°C for 5 min before loading onto the gel. Gels were stained with silver nitrate following the protocol as described in Bassam *et al *[51], and 25 bp DNA marker (Beijing Yuanpinghao Biotech Co., Ltd) was used for calculating the length of EST-SSR amplicons. All primers were detected firstly in *E. sagittatum *to check if the primers can successful amplify PCR products. Then primers succeeding in amplifying bands were tested for transferability. For detecting whether *E. sagittatum *EST-SSRs transferred successfully or not, the PCR products were firstly tested using 2% agarose gel and then polyacrylamide gel.

### Genetic diversity and data analysis

Arlequin [52] was used to calculate the genetic diversity and average allele number. Genetic distance of the SSRs genotype was calculated using the log-transformed proportion of shared alleles ('Dps') by MICROSAT [53]. Polymorphic information content (PIC) was calculated by PIC_CALC http://dl.getdropbox.com/u/695591/PIC_CALC.rar combination with GenAlex6 [54]. A dendrogram was constructed based on Neighbor-Joining method [55] in the PHYLIP package [56].

## Authors' contributions

SZ prepared transcriptome population for sequencing and developed the EST-SSRs and wrote the manuscript. GX cleaned and assembled the raw sequence and carried out GO annotation. JG performed the genotyping of SSR markers. ZF helped with the 454 sequencing and sequence assembly. 454 sequencing was conducted in BAR's lab. YX helped with the collection of *Epimedium *plant materials in the field. YW co-designed and assisted in the manuscript preparation. All authors read and approved the final manuscript.

## Supplementary Material

Additional file 1 **Table S1**. Characterization of *E. sagittatum *ESTs before and after assembly Click here for file

Additional file 2 **Table S2**. Characterization of 18 EST-SSR loci in 52 Epimedium species. Note: ※ indicated these primer pairs transfer in *Epimedium *species with a single band or null. EPS means expected product size for *E. sagittatum*. OPS means observed product size. ND means not determined. Click here for file

Additional file 3 **Table S3**. *Epimedium *species used for detecting the transferability of EST-SSR. Note: *E. koreanum*1, *E. koreanum*2, *E. koreanum*3 indicated that the specimens were collected from Korea, China and Japan, respectively. Click here for file

Additional file 4 **Figure S1**. Dendrogram representing the relationships observed in 52 *Epimedium *species based on 16 EST-SSR markers. *V. hexandra *was used as an outgroup and the distance bar was shown on the bottom of the tree. The number nearby the branch was bootstrap values and the bootstrap value lower than 30% were not shown. Click here for file

Additional file 5 **Figure S2**. Correlation test between consensus annotation percentage and consensus length. Pearson test show that annotation percentage is positively related to consensus length with correlation coefficients 0.96 at the level of 0.01. Click here for file
