# Supplementary material for: Development of a EST dataset and characterization of EST-SSRs in a traditional Chinese medicinal plant, Epimedium sagittatum (Sieb. Et Zucc.) Maxim
Source: BMC Genomics. 2010 Feb 8;11:94. doi: 10.1186/1471-2164-11-94 (PMC2829513; doi:10.1186/1471-2164-11-94)
Supplement: Additional file 1 — Table S1. Characterization of E. sagittatum ESTs before and after assembly [file 1471-2164-11-94-S1.DOC]

**Additional file 1:**

**Table S1 Characterization of *E. sagittatum* ESTs before and after assembly**

| Items | Total length (bp) | Number of sequence reads | Average length (bp) | Maximum length (bp) | Minimum length (bp) | Number of NR match |
| --- | --- | --- | --- | --- | --- | --- |
| Cleaned EST sequence | 50,947,726 | 226,544 | 224.9 | 556 | 50 |  |
| Singlet | 12,306,996 | 59,228 | 207.8 | 556 | 50 | 19,221 |
| Contig | 6,477,110 | 17,231 | 375.9 | 1,911 | 51 | 10,245 |
| Consensus | 18,784,106 | 76,459 | 245.7 | 1,911 | 50 | 29,466 |
