# Supplementary material for: Development of a EST dataset and characterization of EST-SSRs in a traditional Chinese medicinal plant, Epimedium sagittatum (Sieb. Et Zucc.) Maxim
Source: BMC Genomics. 2010 Feb 8;11:94. doi: 10.1186/1471-2164-11-94 (PMC2829513; doi:10.1186/1471-2164-11-94)
Supplement: Additional file 2 — Table S2. Characterization of 18 EST-SSR loci in 52 Epimedium species. Note: ※ indicated these primer pairs transfer in Epimedium species with a single band or null. EPS means expected product size for E. sagittatum. OPS means observed product size. ND means not determined. [file 1471-2164-11-94-S2.DOC]

**Additional file 2:**

**Table S2 Characterization of 18 EST-SSR loci in 52 *Epimedium* species**

| Primer name | SSRs | Forward primer(5'-3') | Reverse primer(5'-3’) | EPS(bp) | OPS (bp) | #alleles | *Ho* | *He* | PIC |
| --- | --- | --- | --- | --- | --- | --- | --- | --- | --- |
| EsESP02 | (GA)7a(AG)7 | AACTCTGAATCTGAACTACACACACC | TCTGGGCTGGCTACTTCACT | 245 | 221-261 | 16 | 0.28 | 0.81 | 0.78 |
| EsESP06 | (AAG)5 | CTCTTTTCCTTTGATCCCCA | CTCTTTCCGCATCCTGAGTC | 259 | 252-272 | 8 | 0.25 | 0.63 | 0.58 |
| EsESP07^※^ | (AAG)5 | CGTTGATGCTCCTACCAAGG | GGAGAGGGATCGGTAGAAGG | 123 | 0 or 123 | ND | ND | ND | ND |
| EsESP08^※^ | (AAG)5 | TGGTGGAGGGAAGAGTTTTG | CCGATCTGGCACTGAAGAAT | 129 | 0 or 129 | ND | ND | ND | ND |
| EsESP09 | (AAG)5 | GCTCCACCTGCTGAAGTACC | GGAGAGGGATCGGTAGAAGG | 142 | 144-272 | 3 | 0.18 | 0.26 | 0.23 |
| EsESP10 | (AAG)5 | TACCCTTGGCATAAACAGCC | CACCAAACACAACAACAGCC | 174 | 164-206 | 6 | 0.04 | 0.37 | 0.35 |
| EsESP11 | (ACA)5 | GCTTCAACACCGACTTGTCA | CAACTTGCAGTGCAGCAGAT | 157 | 150-166 | 5 | 0.3 | 0.61 | 0.53 |
| EsESP12 | (AAG)10 | TGGTCGGTCTGGTGTTGTTA | GAGAAGCTTCGTGAATTGGC | 152 | 119-186 | 16 | 0.6 | 0.9 | 0.89 |
| EsESP13 | (ACA)5 | AACTCAACAACAACCACCACC | TGAGTACAAACCCCACACGA | 145 | 128-164 | 11 | 0.43 | 0.65 | 0.6 |
| EsESP15 | (AG)6 | ATTCCTGGTGGCTCTCCTTT | ACTCCCACCACTGGACTGAC | 265 | 215-286 | 18 | 0.45 | 0.89 | 0.87 |
| EsESP16 | (AG)6 | AACGACTGCCTTTGTGGTTC | TCCCACACTGGACTGACTTG | 211 | 141-232 | 19 | 0.43 | 0.91 | 0.89 |
| EsESP17 | (AG)6 | AATACGCACCAAGGTTCGAC | CAAAGATTTCTACCAAAGCCTCTT | 184 | 123-213 | 17 | 0.49 | 0.9 | 0.88 |
| EsESP18 | (GAA)5 | TGTTATGATGAAGAGGAAGAGGAA | TCCACCACAACACCACAATC | 172 | 106-181 | 27 | 0.48 | 0.92 | 0.91 |
| EsESP21 | (GAA)5 | GCTGTTTTAGGGCATTTGGA | TCCACCACAACACCACAATC | 208 | 158-212 | 23 | 0.35 | 0.94 | 0.93 |
| EsESP22 | (GAA)5 | GCTGTTTTAGGGCATTTGGA | TCTTCTTCTTCTCTCCCCACA | 101 | 91-100 | 4 | 0.04 | 0.17 | 0.17 |
| EsESP23 | (TA)7 | GGAACGAACCGTAAAGCTGA | GTCAGCAAAAGAAAAGGCCA | 260 | 72-136 | 5 | 0.2 | 0.22 | 0.21 |
| EsESP28 | (CAG)5 | CATCACTCTCTTCCTTCGGC | CATGAACGTTGGTTCTGGTG | 129 | 209-292 | 5 | 0.56 | 0.47 | 0.4 |
| EsESP30 | (CAG)6 | GAAACCCCATATCCATCCAA | AAGCATACTCGGCCATTGAC | 185 | 175-194 | 8 | 0.48 | 0.79 | 0.75 |
| Mean | - | - | - | - | - | 11.9 | 0.35 | 0.65 |  |

Note: ※ indicated these primer pairs transfer in *Epimedium* species with a single band or null. EPS means expected product size for *E. sagittatum*. OPS means observed product size. ND means not determined.
