# Supplementary material for: Development of a EST dataset and characterization of EST-SSRs in a traditional Chinese medicinal plant, Epimedium sagittatum (Sieb. Et Zucc.) Maxim
Source: BMC Genomics. 2010 Feb 8;11:94. doi: 10.1186/1471-2164-11-94 (PMC2829513; doi:10.1186/1471-2164-11-94)
Supplement: Additional file 3 — Table S3. Epimedium species used for detecting the transferability of EST-SSR. Note: E. koreanum1, E. koreanum2, E. koreanum3 indicated that the specimens were collected from Korea, China and Japan, respectively. [file 1471-2164-11-94-S3.DOC]

**Additional file 3:**

**Table S3 *Epimedium* species used for detecting the transferability of EST-SSR**

| Number | Species name | Number | Species name | Number | Species name |
| --- | --- | --- | --- | --- | --- |
| 1 | *E. acuminatum* | 20 | *E. hunanense* | 39 | *E. sutchuenense* |
| 2 | *E. baieali-guizhouense* | 21 | *E. ilicifolium* | 40 | *E. truncatum* |
| 3 | *E. baojingense* | 22 | *E. leptorrhizum* | 41 | *E. wushanense* |
| 4 | *E. braychyrrhizum* | 23 | *E. liboense* | 42 | *E. zhushanense* |
| 5 | *E. brevicornu* | 24 | *E. lishihchenii* | 43 | *E. perralderianum* |
| 6 | *E. campanulatum* | 25 | *E. membranaceum* | 44 | *E. grandiflorum* |
| 7 | *E. chlorandrum* | 26 | *E. mikinorii* | 45 | *E. trifoliolatobinatum* |
| 8 | *E. coactum* | 27 | *E. myrianthum* | 46 | *E. alpinum* |
| 9 | *E. davidii* | 28 | *E. ogisui* | 47 | *E. pubigerum* |
| 10 | *E. dewuense* | 29 | *E. pauciflorum* | 48 | *E. diphyllum* |
| 11 | *E. dolichostemon* | 30 | *E. platypetalum* | 49 | *E. perralderianum* |
| 12 | *E. ecalcaratum* | 31 | *E. pubescens* | 50 | *E. pinnatum ssp. pinnatum* |
| 13 | *E. elachyphyllum* | 32 | *E. reticulatum* | 51 | *E. sempervirens* |
| 14 | *E. elongatum* | 33 | *E. rhizomatosum* | 52 | *E. macrosepalum* |
| 15 | *E. epsteinii* | 34 | *E. rupestre* | 53 | *E. Koreanum* 1 |
| 16 | *E. fangii* | 35 | *E. sagittatum* | 54 | *E. Koreanum* 2 |
| 17 | *E. fargesii* | 36 | *E. shuichengense* | 55 | *E. Koreanum* 3 |
| 18 | *E. flavum* | 37 | *E. simplicifolium* |  |  |
| 19 | *E. franchetii* | 38 | *E. stellulatum* |  |  |

Note: *E. koreanum*1, *E. koreanum*2, *E. koreanum*3 indicated that the specimens were collected from Korea, China and Japan, respectively.
