# Supplementary figures and images for: Development of a EST dataset and characterization of EST-SSRs in a traditional Chinese medicinal plant, Epimedium sagittatum (Sieb. Et Zucc.) Maxim
Source: BMC Genomics. 2010 Feb 8;11:94. doi: 10.1186/1471-2164-11-94 (PMC2829513; doi:10.1186/1471-2164-11-94)

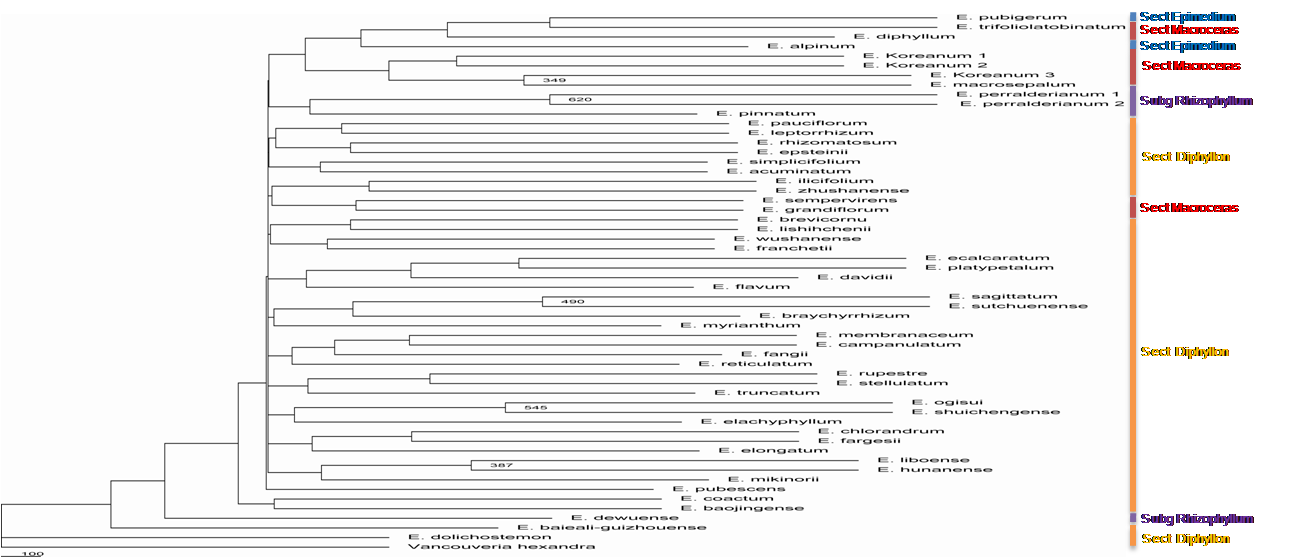

Supplement: Additional file 4 — Figure S1. Dendrogram representing the relationships observed in 52 Epimedium species based on 16 EST-SSR markers. V. hexandra was used as an outgroup and the distance bar was shown on the bottom of the tree. The number nearby the branch was bootstrap values and the bootstrap value lower than 30% were not shown. [file 1471-2164-11-94-S4.TIFF]

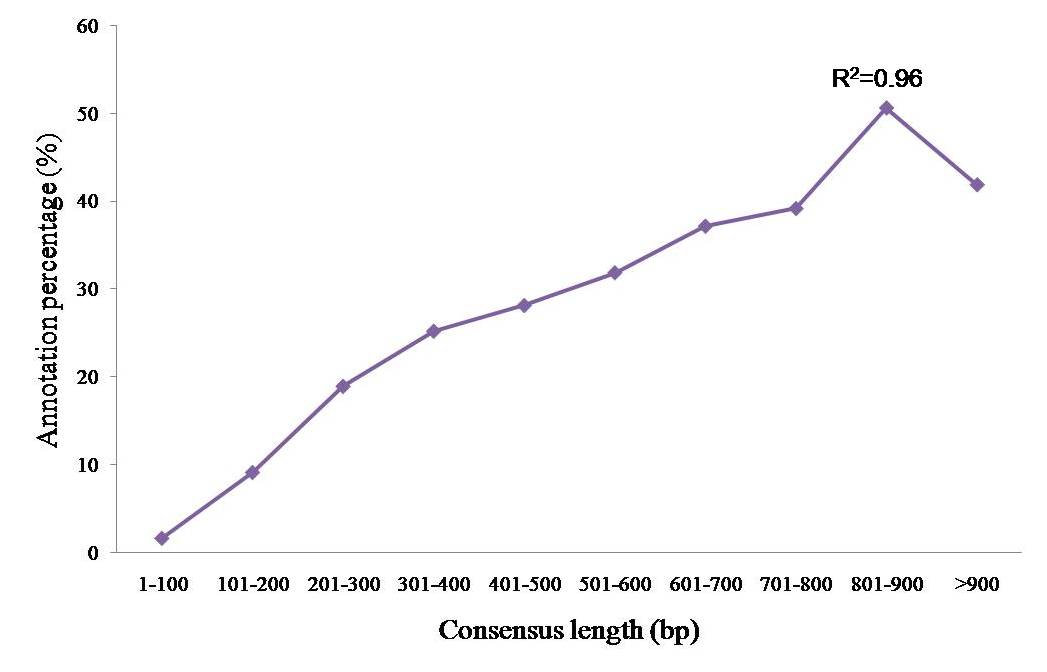

Supplement: Additional file 5 — Figure S2. Correlation test between consensus annotation percentage and consensus length. Pearson test show that annotation percentage is positively related to consensus length with correlation coefficients 0.96 at the level of 0.01. [file 1471-2164-11-94-S5.JPEG]
